# An Unkind Criticism

**Published:** 1885-07

**Authors:** 


### AN UNKIND CRITICISM.

The editor of this journal has evidently been partaking too largely of *Platina*, and has, consequently, formed a somewhat exalted opinion of himself! This egotistical admiration has been suddenly and rudely checked. Instead of *Platina*, we shall hereafter dose ourself with *Aurum*, in order to quickly gather courage for ending our wrecked life by suicide. Our death will lay upon the consciences of Drs. Pope and Dyce Brown, conjoint editors of the world-renowned *Monthly Homœopathic Review!* In commenting on Dr. Wells' *exposé* of the brilliant unfitness of Dr. Dake as a reviser of materia medica, these willful murderers write: "The medium of the attack (*i. e*, the above-mentioned *exposé*) is THE HOMŒOPATHIC PHYSICIAN, an obscure journal published in Philadelphia. We had an opportunity of seeing the first few numbers of it some three or four years ago, but they did not appear to us to foreshadow a periodical that was likely to be of any service, either to medical science in general or to therapeutics in particular."

The only balm to our wounded pride is the hope that *we* have improved somewhat since publishing those fatal "first few numbers," and not gone on year after year publishing stupid nonsense, as has the *Monthly Homœopathic Review.*

